# A double-blind placebo-controlled trial of azithromycin to reduce mortality and improve growth in high-risk young children with non-bloody diarrhoea in low resource settings: the Antibiotics for Children with Diarrhoea (ABCD) trial protocol

**DOI:** 10.1186/s13063-019-3829-y

**Published:** 2020-01-13

**Authors:** The ABCD study team, T. Alam, D. Ahmed, T. Ahmed, M. J. Chisti, M. W. Rahman, A. K. Asthana, P. K. Bansal, A. Chouhan, S. Deb, P. Dhingra, U. Dhingra, A. Dutta, V. K. Jaiswal, J. Kumar, A. Pandey, S. Sazawal, A. K. Sharma, C. McGrath, C. Nyabinda, M. Okello, P. B. Pavlinac, B. Singa, J. L. Walson, N. Bar-Zeev, Q. Dube, B. Freyne, C. Ndamala, L. Ndeketa, H. Badji, J. P. Booth, F. Coulibaly, F. Haidara, K. Kotloff, D. Malle, A. Mehta, S. Sow, M. Tapia, S. Tennant, A. Hotwani, F. Kabir, F. Qamar, S. Qureshi, S. Shakoor, R. Thobani, M. T. Yousufzai, M. Bakari, C. Duggan, U. Kibwana, R. Kisenge, K. Manji, S. Somji, C. Sudfeld, P. Ashorn, R. Bahl, A. De Costa, J. Simon

**Affiliations:** World Health Organisation, Geneva, Switzerland

**Keywords:** Antibiotics, Azithromycin, Paediatric diarrhoea, Mortality, Growth, Randomised

## Abstract

**Background:**

Acute diarrhoea is a common cause of illness and death among children in low- to middle-income settings. World Health Organization guidelines for the clinical management of acute watery diarrhoea in children focus on oral rehydration, supplemental zinc and feeding advice. Routine use of antibiotics is not recommended except when diarrhoea is bloody or cholera is suspected. Young children who are undernourished or have a dehydrating diarrhoea are more susceptible to death at 90 days after onset of diarrhoea. Given the mortality risk associated with diarrhoea in children with malnutrition or dehydrating diarrhoea, expanding the use of antibiotics for this subset of children could be an important intervention to reduce diarrhoea-associated mortality and morbidity. We designed the Antibiotics for Childhood Diarrhoea (ABCD) trial to test this intervention.

**Methods:**

ABCD is a double-blind, randomised trial recruiting 11,500 children aged 2–23 months presenting with acute non-bloody diarrhoea who are dehydrated and/or undernourished (i.e. have a high risk for mortality). Enrolled children in Bangladesh, India, Kenya, Malawi, Mali, Pakistan and Tanzania are randomised (1:1) to oral azithromycin 10 mg/kg or placebo once daily for 3 days and followed-up for 180 days. Primary efficacy endpoints are all-cause mortality during the 180 days post-enrolment and change in linear growth 90 days post-enrolment.

**Discussion:**

Expanding the treatment of acute watery diarrhoea in high-risk children to include an antibiotic may offer an opportunity to reduce deaths. These benefits may result from direct antimicrobial effects on pathogens or other incompletely understood mechanisms including improved nutrition, alterations in immune responsiveness or improved enteric function. The expansion of indications for antibiotic use raises concerns about the emergence of antimicrobial resistance both within treated children and the communities in which they live. ABCD will monitor antimicrobial resistance. The ABCD trial has important policy implications. If the trial shows significant benefits of azithromycin use, this may provide evidence to support reconsideration of antibiotic indications in the present World Health Organization diarrhoea management guidelines. Conversely, if there is no evidence of benefit, these results will support the current avoidance of antibiotics except in dysentery or cholera, thereby avoiding inappropriate use of antibiotics and reaffirming the current guidelines.

**Trial registration:**

Clinicaltrials.gov, NCT03130114. Registered on April 26 2017.

## Background

Acute diarrhoea continues to be one of the most common illnesses in infants and young children, especially in low-income settings. Approximately half a million children continue to die annually as a result of acute diarrhoeal episodes [1], mostly in sub-Saharan Africa or southeast Asia. The current World Health Organization (WHO) recommended management guidelines for acute diarrhoea (rehydration, supplemental zinc, feeding advice and appropriate follow-up) [2] have contributed to significant reductions in diarrhoea-associated mortality [3]. These guidelines do not suggest a role for antibiotics except in case of bloody diarrhoea (as a proxy for shigella or other invasive bacterial infections) or suspected cholera.

Data from the large Global Enteric Multicentre Study (GEMS) [4] show that at least one putative pathogen can be identified in over 80% of children presenting with moderate–severe diarrhoea. Similar results were also seen with a more recent molecular re-analysis of stool samples from children from low-income settings which identified a putative pathogen in 65% of children [5]. A bacterial aetiology was identified in a quarter of all stool samples studied. The recent global burden of disease study on childhood diarrhoea indicates that, while rotavirus is the leading cause of diarrhoea-related deaths (28%) in young children, bacterial species including *Shigella* spp. (14.5%), non-typhoid *Salmonella* spp. (8.4%), *Campylobacter* spp. (9%) and *Escherichia coli* (6.5%) also contribute to a significant number of deaths in this age group [1]. These studies suggest a significant role for bacterial pathogens in diarrhoea-associated deaths [1]. Other studies have also demonstrated that visible blood in the stool is a poor indicator of a bacterial aetiology in children with diarrhoea [6, 7], suggesting that the use of blood in the stool as a proxy for *Shigella* spp. may be inadequate and that the indications for antibiotic use in young children with acute diarrhoea could be expanded.

Taken together, these studies suggest that current treatment guidelines may be missing the opportunity to appropriately provide antibiotics to a highly selected group of young children who, because of aetiology, disease severity and/or undernutrition, are at a particularly high risk of diarrhoea-associated mortality [1, 8, 9]. Azithromycin, a macrolide with a broad spectrum of antibacterial activity and immunomodulatory properties, has been administered to children in the context of mass drug administration programmes largely for trachoma prevention [10]. Observations of mortality sparing related to azithromycin administration in these trachoma interventions prompted several large community trials of azithromycin administered via mass drug administration which have shown significant mortality reductions in children in low-resource settings [11–13].

Given these findings, we are conducting a randomised, placebo-controlled trial, the Antibiotics for Childhood Diarrhoea (ABCD) trial, to determine if the addition of an antibiotic (azithromycin) to the standard management for acute non-bloody watery diarrhoea in a subset of children 2–23 months of age who are dehydrated or undernourished could reduce the mortality and improve growth in settings where such deaths commonly occur.

## Methods

### Aim

The main aim of the ABCD trial is to compare rates of all-cause mortality in the 180 days following enrolment for an episode of acute non-bloody diarrhoea among high-risk children (dehydrated and/or undernourished) aged 2 to 23 months, living in low-resource settings, who are randomised to receive a 3-day course of azithromycin or placebo, in addition to the WHO recommended management of acute watery diarrhoea. A second main aim is to compare the change in linear growth 90 days after enrolment between the same groups.

The secondary aims include a comparison of indicators of acute malnutrition, hospitalisations and/or death. These are described in detail under the section ‘Outcomes’. Given the risk of antimicrobial resistance, we plan to compare resistance profiles in nasopharyngeal (*Streptococcus pneumoniae)* and stool (*E. coli)* isolates from a sample of children from the placebo and treatment arms. The protocol for the main trial is described here.

### Study design

The ABCD trial is a double-blind, individual randomised, parallel group superiority trial comparing azithromycin with placebo conducted in 11,500 high-risk children aged 2–23 months, presenting with non-bloody diarrhoea in seven countries (Bangladesh, India, Kenya, Malawi, Mali, Pakistan and Tanzania).

**Fig. 1:**
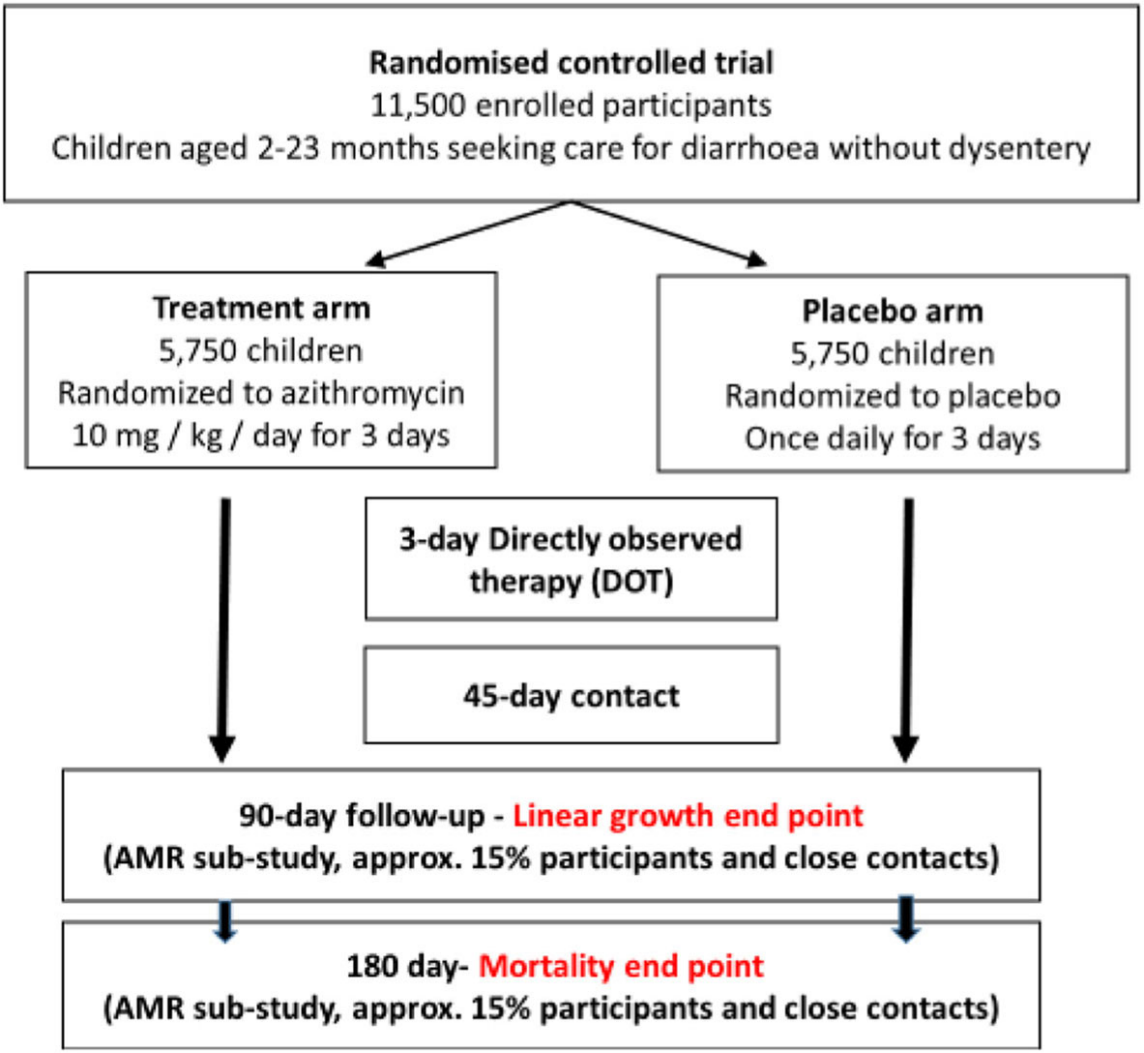
The Antibiotics for Children with Diarrhoea (ABCD) trial study design. AMR antimicrobial resistance

The trial protocol was developed by collaborators at the WHO, Department of Maternal Newborn, Child and Adolescent Health (Geneva, Switzerland) together with teams in Dhaka, Bangladesh (the International Centre for Diarrhoeal Disease Research), New Delhi, India (the Centre for Public Health Kinetics), Nairobi, Kenya (the Kenya Medical Research Institute), Blantyre, Malawi (the Malawi Liverpool Wellcome Trust), Bamako, Mali (the Centre pour le Développement des Vaccins), Karachi, Pakistan (the Aga Khan University), Dar es Salaam, Tanzania (the Muhimbili University of Health and Allied Sciences), Boston, MA, US (the Boston Children’s Hospital and Harvard TH Chan School of Public Health), the University of Maryland, the University of Washington Department of Global Health and the Centre for the Integrated Health of Women, Adolescents and Children. The study design is described in Fig. 1.

### Study setting and population

The study will be implemented in health facilities in the seven sub-Saharan and south Asian countries listed above. The countries were selected based on study site characteristics as well as the strengths and experience of the investigator teams in conducting large intervention trials. Within each country, 2–10 individual health facilities will be the sites of patient enrolment.

### Inclusion criteria

1. Children aged 2–23 months, presenting to a designated health care facility at a participating study site with:
  - Diarrhoea as per caregiver perception and at least three loose or watery stools in the previous 24 h
  - Diarrhoea for less than 14 days prior to screening and with at least one of the following risk criteria at presentation:
    (i) Signs of some or severe dehydration as per the WHO Pocket Book 2013 [14]
    (ii) Moderately wasted as defined by a mid-upper arm circumference (MUAC) <125 mm (but ≥115 mm) or a weight-for-length *z* score (WLZ) greater than −3 and less than or equal to −2 after rehydration during a stabilisation period
    (iii) Severely stunted (length-for-age *z* score (LAZ) less than −3 based on WHO norms)
2. Parent or guardian (caregiver) willing to allow household visits on day 2 and day 3 and willing to return to facility on day 90 post-enrolment
3. Parent or guardian (caregiver) provides written consent for trial participation on behalf of the child

### Exclusion criteria

1. Dysentery (blood in stool reported by caregiver or observed by health care worker)
2. Clinically suspected *Vibrio cholerae* infection
3. Previously or currently enrolled in the ABCD study
4. Concurrently enrolled in another interventional clinical trial
5. Sibling or other child in the household enrolled in the ABCD study and currently taking study medication
6. Signs of associated infections (pneumonia, severe febrile illness, meningitis, mastoiditis or acute ear infection) requiring antibiotic treatment
7. Documented antibiotic use in the 14 days prior to screening (not including standard use of prophylactic antibiotics, i.e. co-trimoxazole use in HIV-exposed children)
8. Documented use of metronidazole within the last 14 days
9. Known allergy or contraindication to azithromycin antibiotics
10. Severe acute malnutrition defined as weight-for-length *z* score less than −3, or MUAC less than 115 mm, or oedema of both feet
11. Living at a distance from the enrolment health centre that prevents adequate directly observed therapy on day 2 and day 3

Site-specific recruitment plans were made. Each site will discuss the protocol and its implementation with medical staff, community health workers in the area and community leaders in order to increase awareness of the trial, as well as to encourage referral of eligible children to enrolling sites.

### Screening and enrolment procedures

After a potentially eligible child has been identified, the study staff will screen the child for eligibility, based on the above-mentioned inclusion and exclusion criteria, using a standardised screening form. If the child presents with diarrhoea and no signs of dehydration, the child is enrolled if anthropometric criteria are met.

If the child has signs of “some” or “severe” dehydration, or if the child requires urgent care, they will be kept under observation. During this “stabilisation” period, oral and/or intravenous rehydration will be provided, and treatment of all urgent conditions will be performed using standard treatment in accordance with the WHO Pocket Book of Hospital Care for Children, 2013 [14]. Assignment to either study drug (azithromycin or placebo) will not require alteration to the current standard of care. Once rehydration of the child is successfully completed, and urgent care has been provided, the child is enrolled, if eligible. However, if the child is not stabilised, or requires additional treatment, they will not be screened further.

Anthropometric measurements will be carried out in accordance with the methods specified in the WHO module on measuring a child’s growth [15]. Anthropometry measurements will be taken after rehydration and stabilisation as required. Weight will be measured using an electronic scale with a sensitivity of ±10 g by two independent observers; length will be measured using a length board to the nearest 0.1 cm. Calibration of the instruments to measure length and weight will be done daily. MUAC will be measured using non-stretchable tape to the nearest 0.1 cm.

### Consent

After confirming eligibility, the accompanying primary caregiver will provide written informed consent. For caregivers who are illiterate, documented witnessed verbal consent and a thumbprint will be obtained. During consent, the purpose of the study, as well as all the study procedures, will be explained to the caregiver by a member of the trial team in the local language.

Consent is taken for the collection and storage of and use of samples collected. Caregivers were informed that they could request discontinuation of storage and destruction of samples collected from the child. Caregivers were also informed that they could choose to participate in the trial but not provide samples.

### Randomisation, allocation concealment and blinding

Stratified randomisation (by site) will be carried out in permuted blocks (block size 4,6,8). A computer-generated randomisation list will be converted into unique serial numbers for each enrolled child at each site. Each individual enrolled child’s supply of study medicine will be provided to each site in advance, labelled with this serial number.

To ensure blinding, the containers and doses for each of the two groups will be identical. The active and placebo medications will be similar in all aspects including the colour, smell and taste. The containers will be identical. Treatment allocation (once assigned) will remain blinded to the participant, the site Principal Investigator, the site study staff and the hospital clinicians during all data collection phases of the study.

The randomisation code is kept with a third-party agency. The code will only be broken if the Data Safety and Monitoring Board (DSMB) requests this information for a participant (because of a suspected relationship between a serious adverse event and the study drug) or for unmasking a study arm (as part of the interim analysis).

### Interventions

Children will be randomised to one of two arms. Those randomised to the treatment arm receive azithromycin 10 mg/kg as a single dose on 3 consecutive days, reconstituted from a dry powder, and given to the children orally. Children randomised to the placebo arm receive an inactive placebo identical in appearance, also as a dry powder, reconstituted similarly, and given for 3 consecutive days.

The first dose of the study medicine is given directly observed at the health facility by a trained study worker. On days 2 and 3, a study team member will visit the home of all enrolled children to provide the subsequent doses of the study medicine, or to observe the caregiver giving it.

Both groups will receive standard of care for diarrhoeal disease, including rehydration, supplemental zinc, nutritional counselling, follow-up and guidance on when to return, as per the WHO guidelines. Children with some or severe dehydration will be rehydrated and stabilised prior to completion of screening.

### Outcomes

#### Primary outcomes

1. All-cause mortality in the 180 days following enrolment between the placebo and treatment arms
2. Change in linear growth measured as change in length-for-age *z* score (∆LAZ) in the 90 days following enrolment between the placebo and treatment arms

#### Secondary outcomes

1. Change in markers of acute malnutrition between arms (∆MUAC and ∆WLZ and ∆weight)
2. The proportion of children who are hospitalised in the 90 days following enrolment
3. The proportion of children who are hospitalised or have died in the 90 days following enrolment
4. The proportion of children who are hospitalised or have died in the 10 days following enrolment
5. Cause-specific mortality as determined by verbal and social autopsy
6. Proportion of strains of *E. coli*, isolated from stools, resistant to azithromycin and other antibiotics at enrolment among the study population
7. Proportion of strains of *E. coli*, isolated from stools, and *S. pneumoniae*, isolated from nasopharyngeal swabs, resistant to azithromycin and other antibiotics at day 90 and day 180 in a randomly selected sub-sample of children enrolled in the study and their siblings or close household contacts

### Trial assessments and follow-up

**Fig. 2:**
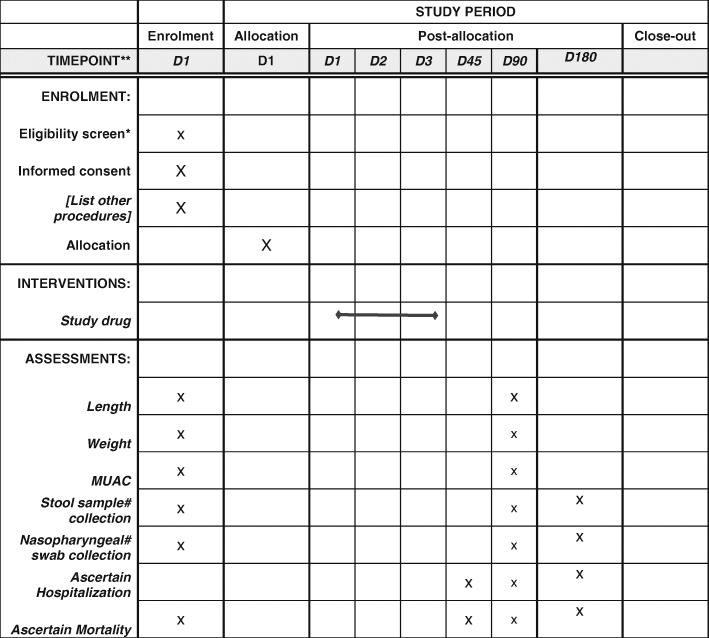
Schedule of enrolment, intervention and follow-up assessments for the Antibiotics for Children with Diarrhoea (ABCD) trial *Screening is expected to be completed on the same single day, except is the child needs treatment for dehydration or urgent care for an illness, in which case, it can be completed once the child has been stabilized. #Stool samples and nasopharyngeal isolates are only collected for 15% of participants in the antimicrobial resistance study. D=day. MUAC=mid-upper arm circumference

Children enrolled into the trial will be followed up for 180 days post-enrolment or until the first primary end point is reached, whichever is earlier. The schedule of enrolment, intervention and follow-up assessments is shown in Fig. 2, which follows the SPIRIT guidelines.

At day 45, a telephonic or in-person contact will be made by a non-medical member of the trial team to ascertain the vital status of the child, any hospital admissions and to remind the patient of the day 90 appointment.

At day 90, participants will be followed-up in the clinic to ascertain vital status, hospitalisations, and health of the enrolled child, as well as their nutritional status (weight, length and MUAC).

Participant retention will be encouraged by the frequent follow-up during days 1–3, the provision to caretakers of a study telephone number for questions, and the follow-up schedule noted in Fig. 2. Caretakers of enrolled children provided study staff with their home address and the closest available telephone number on which they could be contacted. At day 180, study personnel will contact study participants by telephone or in person. They will ascertain the vital status of the enrolled child at day 180 and document any hospital admissions since the day 90 visit.

If a child is reported to have died at any time during the study follow-up period, specially trained study staff will review the hospital record (if available) and conduct a standardised verbal and social autopsy interview to ascertain the date, cause and context of death. The verbal autopsy effort will include capturing all relevant hospital/facility-based information on a child that dies in a facility.

### Antimicrobial resistance

A subset of 1700 ABCD participants will be enrolled in an antimicrobial resistance (AMR) detection study in the trial. This AMR component will study the development of AMR among *E. coli* (stool isolates) and *S. pneumoniae* (nasopharyngeal isolates) in study participants and their close household contacts.

A faecal stool sample will be collected from all children at enrolment for culture and sensitivity testing of *E. coli*.

Children enrolled in the AMR sub-study will also be asked to provide a stool sample and a nasopharyngeal swab on day 90 and day 180. In addition, a stool sample and a nasopharyngeal swab will also be collected from a sibling or other close household contact of the enrolled child at day 90 and day 180. The protocols for this AMR detection study will be described elsewhere.

### Diarrhoea aetiology

Each site will undertake an assessment of the potential viral and bacterial pathogens associated with the diarrhoeal episode in a separate study. A total of 7000 stool specimens will be tested. Identification will be facilitated by quantitative molecular diagnostic methods.

### Recording of serious adverse events

Serious adverse events are defined as any deaths or hospitalisation or any life-threatening event that occur in the period from enrolment to day 10. Although it is relatively safe for use in young children, azithromycin can cause adverse events including nausea, abdominal pain [16] and diarrhoea from its effects on the gut microbiota [17, 18]. Any adverse events in the ABCD trial are monitored by the Trial Steering Committee (TSC) and reviewed by the DSMB. These events will be recorded by the trial staff and confirmed by the study physicians. Study physicians can, at any time, withdraw a participant from the trial if a risk to their safety from continuation in the study is perceived.

### Quality assurance

The following measures provide quality assurance:

1. An extensive initial and subsequent ongoing training sessions for data collectors in the protocol procedures with a focus on anthropometry measurements and dehydration assessment to ensure the reliability between data collectors is high.
2. Real-time electronic data capture will ensure data validation, such as range, logical checks and data integrity.
3. Principal Investigators will receive brief monthly progress reports from the Data Coordinating Centre during the entire study period and will participate in regular telephone conferences with WHO staff. The monthly progress reports will include the number children assessed, number of children recruited, home visits due to be conducted, actual visits conducted, child hospitalisations, deaths and verbal and social autopsies [19] conducted.
4. Field supervisors will be responsible for assuring that the training of the field staff is rigorous and of high quality. They will schedule the testing and retraining as required at their individual sites. Assessment of individual study personnel’s abilities to use the standardised enrolment criteria and conduct the anthropometric measurements and dehydration assessment consistently across the study population are key responsibilities of the field supervisor.
5. WHO study coordinators, and others identified by them, will ensure that at least two structured monitoring visits are conducted to each site every year. The monitoring visits will have as their primary aim quality control and the improvement of study implementation. The monitors will make direct observations of all relevant study procedures and data management activities.
6. The data management team will run a monthly set of range and consistency checks, resolve inconsistencies or queries with the sites and provide data summaries as the trial progresses. The queries will be resolved before the next set of monthly checks report.

### Compensation

No financial compensation will be provided for participation in the trial. The presenting diarrhoeal episode and any serious illnesses will be treated at the time of presentation.

### Data management

An external data management agency will ensure harmonisation of data collection and data management processes across all sites. All sites will collect information on a core set of variables with standard definitions. A set of range and consistency checks that must be applied to these variables will be available.

Each site will be responsible for data entry and initial cleaning of the data, including running range and consistency checks, as well as periodic reviews of distributions and identification of outliers. Each study site will resolve any inconsistencies within its database, in consultation with the field data collection team, and with field verification if needed. Individual sites will be required to provide data on the core set of variables into a REDCap database. The data management team will run a monthly set of range and consistency checks, resolve inconsistencies or queries with the sites and provide data summaries as the trial progresses.

### Statistical considerations

#### Sample size and power

To determine the sample size for the ABCD trial, we estimated that the 180-day mortality in the control group would be 2.7%. The estimated overall mortality in the control group was based on the earlier GEMS trial [4] as well as the assumed proportions of children with various risk factors in the sample and respective sub-group-specific mortality. Assuming this baseline mortality, a relative risk in the intervention group of 0.65 (35% reduction in mortality in the intervention group), 90% power, 95% confidence, assumed loss to follow-up of 10%, and 1:1 ratio in the numbers of participants in the control and intervention group, the required sample size would be 5696 per group or 11,392 in total. Since there is an initial plan to conduct one interim analysis, the sample size will be inflated by a factor of 1.009 [20]. The total planned sample size will be 5750 per group and 11,500 in total. As the study is implemented in seven countries, each country will on average need to enrol 1645 children (approximately 822 per study group).

The second primary aim is to compare ∆LAZ between the control and intervention from enrolment to 90 days. We have estimated that, with the above sample size, we will have 80% power to detect at least a 0.04 difference in mean ∆LAZ between study groups using a two-sided *t* test to compare the difference in mean change in LAZ between two groups with α = 0.05 and standard deviation (SD) of 0.7 in both groups. This is comparable to results observed in GEMS. As the SD of the difference in ∆LAZ has been shown to vary, this will provide an adequate sample size to detect a ∆LAZ ranging between 0.04 and 0.06 given varying SDs between 0.5 to 0.7, with a power of 80–90%.

#### Statistical analysis

An intention-to- treat (ITT) approach, including all randomised participants, is considered the primary analysis approach.

For the first primary outcome, i.e. the mortality outcome, period prevalence of death will be compared between the randomly assigned treatment groups using relative risk regression. Mortality (a binary variable) will be defined as any event of death from time of randomisation to end of day 180. Participants with a missing mortality outcome will be assigned ‘alive’. For the primary ITT analysis, the following model will be used:

$$ \boldsymbol{E}\left(\boldsymbol{Y}|\boldsymbol{randomisation}\ \boldsymbol{arm}\right)={\boldsymbol{e}}^{\left({\boldsymbol{\beta}}_{\mathbf{0}}+{\boldsymbol{\beta}}_{\mathbf{1}}{\boldsymbol{X}}_{\boldsymbol{azm}}\right)} $$

where x_*azm*_ is an indicator variable specifying randomised to the azithromycin group (x_*azm*_ = 1) or not (x_*azm*_ = 0). The risk ratio comparing the risk of death in children randomised to azithromycin versus placebo will be determined by *e*^*β*^. The statistical significance of this comparison will be determined by a Wald test. In a per-protocol analysis, secondary to the ITT analysis, relative risk regression will be fit as described above among the subset of children with documented completion of the full course of treatment.

For the co-primary outcome, i.e. ∆LAZ, a linear regression model will be used to compare mean ∆LAZ across the treatment groups in surviving children. ∆LAZ will be operationalised as the difference in LAZ between the 90-day follow-up visit and LAZ at baseline. For the primary ITT analysis, LAZ outcome will only be analysed for those children with a measured outcome. The following model will be used:

$$ E\;\left(Y| randomisation\; arm\right)={\beta}_0+\beta {X}_{azm} $$

where Y = mean ∆LAZ, and x_*azm*_ is an indicator variable of being randomised to the azithromycin group (x_*azm*_ = 1) or not (x_*azm*_ = 0). The mean difference in ∆LAZ in children randomised to azithromycin versus placebo will be determined by (*β*). The statistical significance of this comparison will be determined by independent *t* test. In a per-protocol analysis, also secondary to the ITT analysis, a linear regression model will be fit as described above among the subset of children with documented completion of the full course of treatment.

A secondary analysis will be done as a per-protocol analysis excluding participants who are not adherent to the full treatment schedule or having missing outcomes.

Effect modification by site, age, sex, anthropometry and socioeconomic status will be explored. This is planned to be exploratory. No testing within any stratum is envisaged as there is unlikely to be adequate power to test within a stratum.

### Trial governance

The ABCD trial is overseen by the TSC which consists of the site Principal Investigators and the WHO Trial Coordinators. The TSC is responsible for overall supervision of the trial. A second committee, the Trial Advisory Group (TAG), consists of external experts with experience in diarrhoeal disease in low-income contexts. The TAG provides advice to the TSC periodically during the life of the trial.

An independent DSMB has been established by the WHO to monitor severe adverse events and to approve the statistical analysis plan and associated stopping rules for benefit, futility, or harm determined using O’Brien-Fleming stopping boundaries. The DSMB includes five members with expertise in clinical trials, statistics, child mortality assessment, ethics, and paediatric care in resource-limited settings. When approximately half of the person-time is accrued in the study, the DSMB will review an interim data analysis by arm to determine whether stopping boundaries have been crossed. The SPIRIT checklist for present study is provided in Additional file 1.

## Discussion

ABCD is a large, multi-site, paediatric trial testing the potential benefits of azithromycin in reducing mortality and improving linear growth when targeted to high-risk children with non-bloody diarrhoea. A recently concluded cluster randomised trial of mass drug administration of azithromycin has shown a reduction in all-cause mortality among children [11–13]. However, the effect appeared limited to a single site in this trial. Another large trial of azithromycin in addition to seasonal malaria chemoprophylaxis [21] has not documented any benefit on mortality, although a reduction in gastrointestinal infections was seen. The potential effect of azithromycin on mortality in children with a high risk of a diarrhoea-related death has not been studied to date.

Young children who are undernourished or have dehydrating diarrhoea are at higher risk of death in the 3-month period after the onset of diarrhoea [4]. More recently, global data suggest that multiple bacterial and parasitic diarrhoeal pathogens are significantly associated with death, particularly in sub-Saharan Africa [1]. In low -resource settings, the high burden of bacterial causes of diarrhoea [1, 3, 5] has led to suggestions that antibiotics be used more widely, even in the absence of dysentery, as most children with these infections do not have bloody stools [6, 7].

Despite WHO recommendations for the management of diarrhoea which suggest that only children with bloody diarrhoea receive antibiotics, over 40% of children with non-bloody diarrhoea currently receive antibiotics as part of non-standard treatment in low-income settings [22]. This overuse of antibiotics contributes to the development and spread of antimicrobial resistance, both at individual and population levels. While concerns have been raised about expanding the use of empiric antibiotic treatment for diarrhoea, targeting such treatment to a specific sub-group of vulnerable children at high risk of death may potentially lower antimicrobial resistance if prescribers perceive that specific sub-groups of children at the highest risk are being targeted with antibiotic treatment. Prior studies in pneumonia have documented reduced antibiotic use when guidelines were updated to clarify which risk groups should be treated [23]. The ABCD trial will determine if providing azithromycin to children with acute diarrhoea who are at risk reduces mortality and/or diarrhoea-related morbidity, including linear growth faltering. These data will inform the global debate regarding the potential role of antibiotics in reducing child mortality.

If the ABCD trial demonstrates benefit, this will provide evidence to support a reconsideration of the present WHO guidelines for the management of diarrhoea in a clearly identified high-risk population. Conversely, if there is no evidence of benefit, a convincing case for more rigorous control over the inappropriate use of antibiotics in diarrhoeal case management and further strengthening of global efforts to expand coverage and improve coverage of the current Integrated Management of Childhood Illness guidelines can be made.

## Trial status

Recruitment to the ABCD trial began in December 2017 and is currently ongoing. It is expected to conclude in March 2020. The current protocol is version 9.0 and is dated 21 December 2018.

## Supplementary information

**Additional file 1.** SPIRIT Checklist for the ABCD trial.

## Data Availability

The datasets generated during the current study are available from the corresponding author on reasonable request.
